# Comparative Hazard Identification by a Single Dose Lung Exposure of Zinc Oxide and Silver Nanomaterials in Mice

**DOI:** 10.1371/journal.pone.0126934

**Published:** 2015-05-12

**Authors:** Ilse Gosens, Ali Kermanizadeh, Nicklas Raun Jacobsen, Anke-Gabriele Lenz, Bas Bokkers, Wim H. de Jong, Petra Krystek, Lang Tran, Vicki Stone, Håkan Wallin, Tobias Stoeger, Flemming R. Cassee

**Affiliations:** 1 Centre for Sustainability, Environment and Health, National Institute for Public Health and the Environment, Bilthoven, The Netherlands; 2 Heriot-Watt University, School of Life Sciences, Nanosafety Research Group, Edinburgh, United Kingdom; 3 National Research Centre for the Working Environment, Copenhagen, Denmark; 4 German Research Center for Environmental Health (GmbH), Institute of Lung Biology and Disease, Helmholtz Zentrum München, Neuherberg, Germany; 5 Centre for Safety of Substances and Products, National Institute for Public Health and the Environment, Bilthoven, The Netherlands; 6 Centre for Health Protection, National Institute for Public Health and the Environment, Bilthoven, The Netherlands; 7 Philips Innovation Services, Eindhoven, The Netherlands; 8 IOM, Edinburgh, United Kingdom; 9 Department of Public Health, Copenhagen University, Copenhagen, Denmark; 10 Institute for Risk Assessment Studies, Utrecht University, Utrecht, The Netherlands; French National Centre for Scientific Research, FRANCE

## Abstract

Comparative hazard identification of nanomaterials (NMs) can aid in the prioritisation for further toxicity testing. Here, we assessed the acute lung, systemic and liver responses in C57BL/6N mice for three NMs to provide a hazard ranking. A silver (Ag), non-functionalised zinc oxide (ZnO) and a triethoxycaprylylsilane functionalised ZnO NM suspended in water with 2% mouse serum were examined 24 hours following a single intratracheal instillation (I.T.). An acute pulmonary inflammation was noted (marked by a polymorphonuclear neutrophil influx) with cell damage (LDH and total protein) in broncho-alveolar lavage fluid (BALF) after administration of both non-functionalised and functionalised ZnO. The latter also induced systemic inflammation measured as an increase in blood neutrophils and a decrease in blood lymphocytes. Exposure to Ag NM was not accompanied by pulmonary inflammation or cytotoxicity, or by systemic inflammation. A decrease in glutathione levels was demonstrated in the liver following exposure to high doses of all three nanomaterials irrespective of any noticeable inflammatory or cytotoxic effects in the lung. By applying benchmark dose (BMD) modeling statistics to compare potencies of the NMs, we rank functionalised ZnO ranked the highest based on the largest number of affected endpoints, as well as the strongest responses observed after 24 hours. The non-functionalised ZnO NM gave an almost similar response, whereas Ag NM did not cause an acute response at similar doses.

## Introduction

The potential for consumer and occupational exposure will rise with increasing production of nanomaterials (NMs). Therefore, there is a need to consider the possibility of detrimental health consequences of these man-made NMs. The health risk should be assessed based upon the level of exposure to the engineered NM, the toxicity of the material in question (hazard identification) and the route of exposure. The lungs are in constant contact with the external environment and are believed to be the most important route of exposure to NMs [1].

Here, we focus on the hazard identification of *in vivo* acute effects after 24 hours after a single intratracheal instillation (I.T.) of three selected NMs (non-functionalised ZnO, functionalised ZnO and a suspended silver NM). These NMs are available in the JRC NMs repository and are examples of commercial materials used in various applications [2, 3]. The NMs have been extensively characterised within the European Commission (FP7) funded consortium named Risk Assessment of Engineered Nanoparticles (ENPRA, www.ENPRA.eu). Primary particle size, shape, surface area, surface chemistry such as coatings and agglomeration state amongst others prior to administration of the materials have been determined [4]. Within this consortium, seven additional NMs have been characterised, including five types of titanium dioxide and two types of multiwall carbon nanotubes. The Ag and ZnO NMs were selected for *in vivo* studies based on a strong reduction in cell viability (compared to the other materials) observed in hepatocytes and renal cells [4, 5] as well as in LA-4 epithelial cells and MH-S alveolar macrophages (S1 Fig). A commonly used healthy mouse model (C57BL6) was chosen for the entire EU project that also allowed a comparison with other studies within this project using a genetically modified strain on a C57BL6 background.

It is known that NMs administered via instillation or inhalation can translocate from the lung to the circulation and eventually reach secondary tissues [6, 7]. Other studies have demonstrated that after inhalation of 133 μg/m^3^ of nano-silver for 6 hours, a small amount was detected in the liver, kidney, spleen, brain, and the heart in rats [8]. Therefore, in the present study the acute lung effects based on markers of cell damage and inflammation in the broncho-alveolar lavage fluid (BALF), as well as responses in the systemic circulation and the liver were investigated. The liver, the metabolic centre of the body, has been shown to accumulate NMs at higher concentrations to other distal organs [8–12]. Some NMs are known to generate reactive oxygen species (ROS) *in vivo*, either by direct chemical reactions at their surface [13–16], intracellularly [5, 16, 17] or by indirect processes related to an immune response [18]. ROS generation may impair the antioxidant defence system leading to oxidative stress and cytotoxicity [14]. Glutathione is an antioxidant that scavenges ROS molecules and prevents oxidation of protein sulfhydryl groups [19]. Alterations in glutathione levels can be indicative of acute oxidative impact on the liver. Therefore, the glutathione content in this organ was evaluated within this study.

We used I.T. instillation as a comparative method for hazard identification, since it is easier to control the administered dose compared to inhalation [20]. For the comparison of potencies of the various NMs (within an endpoint), benchmark dose (BMD) modelling is applied. The derived BMD is an equipotent dose, which means that the dose that gives the same effect level e.g. a 10% increase in LDH levels compared to the control is determined for each nanomaterial. This facilitates an accurate comparison of the different nanomaterials used here [21, 22].

## Materials and Methods

### Nanomaterials

The nanomaterials in this study have been commissioned by the European Commission Joint Research Centre repository (JRC, Ispra, Italy): NM-110 ZnO (non-functionalised, 100 nm), NM-111 ZnO (functionalised with triethoxycaprylylsilane, 130 nm), NM-300 Ag (capped with polyoxylaurat Tween-20, <20 nm). The NMs were sub-sampled and preserved under argon in the dark at room temperature until use. The details on raw material characteristics, such as primary particle size as stated by the manufacturer, X-ray diffraction (XRD) size and transmission electron microscopy (TEM), surface area according to Brunauer Emmett Teller (BET) (only applicable for ZnO dry powders) and coating have been described previously [4] and are briefly summarized in S1 Table.

### Nanomaterial suspensions

NM stock suspensions were prepared in MilliQ water supplemented with 2% volume of house-prepared isogenic mouse serum at a concentration of 2.56 mg/ml. Functionalised and non-functionalised ZnO samples were prewetted with 0.5 vol.% of 96% EtOH before the addition of MilliQ water and sonication for two times 8 minutes on ice at an amplitude of 10% using a Branson Sonifier S-450D (400 W, Branson Ultrasonics Corp., Danbury, USA). The Ag NM was supplied as a liquid with 10% w/w (100mg/ml) Ag in de-ionised water (75%) with 7% ammonium nitrate as the stabilizing agent and 8% emulsifiers (4% each of polyoxyethylene glycerol trioleate and polyoxyethylene (20) sorbitan mono-laurat (Tween 20)). The stock suspensions were stable for at least 1 hour. All dilutions were prepared as quickly as possible and sonicated for 1 minute before use.

Particle size distributions were determined directly before intratracheal instillation. A dilution of 20 μg/ml from the stock suspension was used for 5 separate measurements utilising tracking analysis of Brownian motion with a laser illuminated microscope (LM20, NanoSight Ltd, UK). The particle suspensions and mouse serum were tested in a Limulus Amebocyte Assay (kinetic turbidimetric LAL assay). The nanomaterials did not contain any detectable endotoxin, while the mouse serum contained a very low amount of 2 IU/ml. This corresponds to 0.002 IU of endotoxin per mouse instillation and is unlikely to have an effect.

### Animals

Healthy female C57BL/6NTac inbred (murine pathogen free) mice of 7–8 weeks of ~20 grams (18.1–22.7 grams) were obtained from Taconic (Ry, Denmark). After arrival, the animals were weighed and randomly allocated in groups of 10 in Macrolon II cages. Animals were kept in a controlled specific pathogen-free environment, fed standard rodent chow ad libitum, under a 12h day/ 12h night rhythm. The animals were allowed to acclimatize for 1–2 weeks before starting the experimental protocol and were monitored daily for general health.

### Ethics Statement

The animal experiments are conducted according to all applicable provisions of the Dutch national law: Experiments on Animals Act from 1997. This study was approved by the Animal Experimentation Ethical Committee of the National Institute for Public Health and the Environment in Bilthoven, the Netherlands under permit number 201000023.

### Intratracheal instillation

The mice were taken from the home cage and anesthetized using 4% isoflurane in an induction chamber. The anaesthesia was maintained using 2.5% isoflurane via an intubation aid (UNO BV, The Netherlands) during I.T. instillation as previously described [23]. In short, a diode light was placed against the larynx, the tongue was pressed towards the lower jaw and the trachea was intubated using a 22 gauge catheter with a shortened needle. A 50 μl suspension of the NMs was instilled followed by 200 μl of air. The vehicle control mice received the sonicated dispersion media with 2% serum without NMs. For both ZnO NMs, this included 0.5% ethanol in MilliQ water with 2% mouse serum. For the Ag NM, the vehicle control was the Ag dispersant with 2% serum. The animals were dissected 24 hours after instillation.

The study set-up for all NMs included three animals per group and a dose-range of 0, 1, 4, 8, 16, 32, 64 and 128 μg/mouse. Instilled doses of NMs up to 200 μg/mouse [24] and 168 μg/mouse [25] have been used before. Additional animals (5 per group) received a dose of 0, 1, 8, 32, 64 and 128 μg/mouse non-functionalised ZnO. Their lungs were flushed for broncho-alveolar lavage fluid (BALF) analysis and subsequently prepared for pathology.

### Lung analysis

Lungs were lavaged twice using 1 ml saline per 25 g body weight. Each flush consisted of three slow up and down movements. The BALF was centrifuged at 400g at 4°C for 10 minutes with a range of proteins analysed in the supernatant. These included a panel of 11 cytokines: interleukin-1β (IL-1β), tumor-necrosis factor-α (TNFα), interleukin-6 (IL-6), granulocyte-colony stimulating factor (G-CSF/CSF3), IL-8 like keratinocyte chemo-attractant (KC/CXCL1), monocyte chemotactic protein-1 (MCP-1/CCL2), interleukin-4 (IL-4), interleukin-13 (IL-13), interleukin-12 (IL-12p40), macrophage inflammatory protein-1β (MIP1β/CCL4), and RANTES/CCL5 (Bio-Plex Pro Mouse Cytokine 11-Plex Panel, #M60006RDAY, BioRad, Munich, Germany). Furthermore, the lactate dehydrogenase (LDH), alkaline phosphatase (ALP), albumin and total protein were measured using test kits and an auto-analyser (LX20-Pro, Beckman-Coulter, Woerden, The Netherlands). The cell pellet was suspended in 500 μl for cell counts (Coulter counter) and cell differential counts of 400 cells (cytospins). In a total of 5 out of 102 lung lavages, the BALF was not analysed. This is indicated in the raw data file as NA for not analysed (S1 dataset). In 1 animal, the lavage was accidentally not performed and in the other 4 animals fluid was lost due to leakage of the lungs which renders the obtained results unreliable.

Lungs of mice (n = 5) were fixed in formalin before being embedded in paraffin. For the control animals and the two highest dose-groups slides were prepared which were representative of three slice sections through the lung. After standard histochemistry processing and haematoxylin and eosin (H&E) staining, the slides were examined by light microscopy.

### Blood analysis

EDTA blood was used to determine the total number of cells, cell differential count (erythrocytes, neutrophils, lymphocytes and platelets) and haemoglobin content using test kits and an auto-analyzer (LX20-Pro, Beckman-Coulter, Woerden, the Netherlands).

### Liver analysis

The mouse liver samples were thawed on ice and homogenized in 2 ml of lysis buffer on ice and centrifuged (700g for 5 minutes). The tissue was re-suspended in ice-cold buffer [26], mixed and incubated on ice for 10 minutes before being centrifuged at 15000 g for 5 minutes to generate lysates and protein pellets. The glutathione content of the lysate was quantified by reaction of sulfhydryl groups with the fluorescent substrate *o*-phthaladehyde (OPT) using a fluorometer λ_ex_ = 350 nm and λ_em_ = 420 nm. The protocol was slightly modified to include measurements of total glutathione by reducing oxidized glutathione dimers (GSSG) by addition of 7 μl of 10 mM sodium dithionite to all samples and incubating at room temperature for 1 hour. The values were corrected for the total amount of protein.

The baseline Ag content in liver tissue was determined in the vehicle control mice, 64 and 128 μg/mouse dose groups by High Resolution Inductively Coupled Plasma Mass Spectrometry (HR-ICPMS; ELEMENT XR, Thermo Fisher Scientific, Germany). In short, ~ 500 mg of the left liver lobes (fixed in formaldehyde) were transferred into digestion tubes. A volume of 2 ml of nitric acid, 60% (HNO_3_) was added for the complete digestion of the sample material. The mixture was kept at a maximum temperature of 120°C overnight. The digests were diluted prior to analysis by HR-ICPMS. Silver was measured as ^107^Ag; while ^109^Ag was used for control. As there was no interference for both isotopes, low resolution mode was selected for maximal sensitivity. For the quantification, external calibration with internal standard correction by rhodium as ^103^Rh was carried out. The Ag background concentrations from the used chemicals and contact materials were calculated. Within the same sequence, the certified reference material “DOLT-4 Dogfish Liver certified material for trace metals” (NRC CNRC, Canada) was analysed. Silver in this reference material is certified as a total mass fraction of (0.93 ± 0.07) μg Ag/g. Our result (n = 6) of 0.80 ± 0.05 μg Ag/g is in sufficient agreement to suggest that the technique used was both reliable and accurate.

### Study design and statistics

Dose-response relationships were analysed using dose-response modelling software (PROAST version 23.9 for continuous and version 30.4 for quantal datasets; software is freely available at www.rivm.nl/proast). Models are fitted to the data, a benchmark response (BMR) is defined, and the associated BMD is derived from the fitted model [27, 28]. The instillation study design included a minimum of 6 dose groups which incorporated controls with 3 animals per group (or incidentally 2 animals due to non-analysable BALF, see S1 dataset), which is suitable for this type of statistical analysis. By applying this type of statistics with 6 dose groups and 3 animals per group, instead of 3 dose groups with 6 animals per group, it is more likely to find a dose which leads to a response and reduces the total number of animals for a hazard identification study.

A BMR of 10% change in response was chosen for the analysis of the total number of cells, protein, LDH and cytokine levels. The total number of macrophages, lymphocytes, eosinophils and neutrophils (in the BALF) cannot be analysed, because only a limited (total) number of 400 cells were counted. The uncertainty of each cell count (and percentage) depends on the total number counted. Extrapolating these counts to the total number conceals the uncertainty. To avoid this, the counts were analysed as clustered quantal data, i.e. as a number within a sample of 400 cells, where the BMR was set to 10% extra risk [29]. Optimal models from the exponential and Hill model families were selected according to the likelihood ratio test [28]. The 90%-confidence interval (CI) surrounding this BMD comprises of the 5% lower confidence bound (BMDL) and the 95% upper confidence bound (BMDU) found for the BMD estimates derived from the different models. It should be noted that the BMD approach is applied here to assess the differences in potency between nanomaterials and not to derive a point of departure (i.e. a BMDL) for further risk assessment. Comparing potencies requires the best estimate of the BMD, i.e. the BMD itself, and not the BMDL [21, 22]. The confidence interval surrounding the BMD is only used to assess the quality of the derived BMD. To determine whether BALF results from mice receiving non-functionalised ZnO material (including 5additional animals for pathology in which BALF analysis was also performed) were different, a likelihood ratio test [28] was performed. The results were not significantly different from each other and therefore, the data was pooled to derive a single BMD. Therefore, a BMD for total cell counts, neutrophils, IL-6, GCSF, LDH and protein for non-functionalised ZnO is derived from data of 8 mice (or incidentally 7 mice due to non-analysable BALF, see S1 dataset).

## Results

### Particle characteristics

Primary and aggregate particle sizes in suspension were determined by various analytical techniques [4] (S1 Table). Electron microscopy (EM) analysis revealed two morphologies of ZnO crystallites with different size ranges. XRD analysis revealed the crystallite size for non-functionalised and functionalised ZnO to be 70–100 nm and 58–93 nm, respectively. TEM analysis of the Ag NMs showed the particles to be between 8 and 47 nm. The size distribution of all materials, freshly prepared in their dispersion media containing 2% serum, was measured just before I.T. instillation. Previously, we have shown that in the presence of serum, suspensions are stable for at least one hour [4]. NM size distributions around 200 nm for all particle suspensions indicate that there was some degree of agglomeration in the presence of serum after sonication. The mode and mean NM size were not similar suggesting an asymmetrical particle size distribution (S2 Table).

### Acute lung responses—ZnO

**Fig 1:**
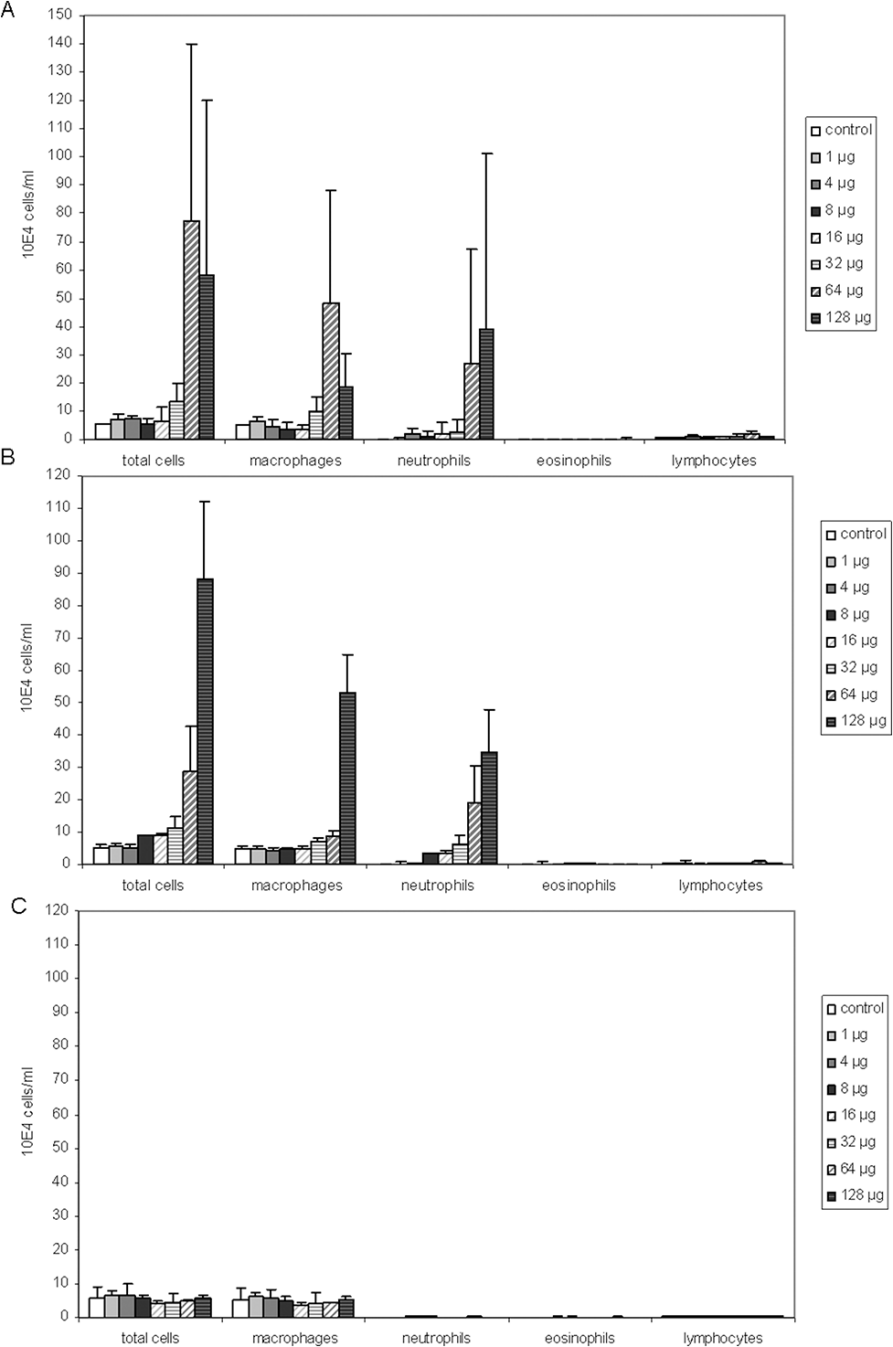
Differential cell counts in BALF. Average total cell numbers and macrophages, neutrophils, eosinophils and lymphocytes (n = 3), 24 hours after nanomaterial instillations with A) non-functionalised ZnO, B) functionalised ZnO and C) Ag NMs. The dose is the representative of μg of NM instilled per mouse. Error bars represent the standard deviation.

The cellularity, cytokine responses and cell damages markers were analysed in the BALF, 24 hours after NM administration. For both ZnO NMs, an increase in total number of BAL cells was observed after administration of the two highest doses of 64 and 128 μg/mouse, indicative of an inflammatory response (Fig 1A and 1B). This increase was attributed to a rise in the number of macrophages and neutrophils. The data has been analysed using BMD modelling and the dose of functionalised ZnO exposure required to cause a 10% increase (BMD10) in total cell numbers compared to the vehicle control, was 9.4 μg/mouse (confidence interval (CI) = 7.1–64.0 μg/mouse). In comparison, the BAL cell number BMD10 for the non-functionalised ZnO was 29.5 μg/mouse (CI = 23.7–55.6 μg/mouse). The lower BMD10 indicates that the functionalised material was more potent than the non-functionalised ZnO. However, based on the overlapping confidence intervals, the potencies of both ZnO NMs to increase BAL cell numbers were not significantly different (Table 1). The non-functionalised ZnO was marginally more potent in inducing a neutrophilic response (BMD10 = 2.8, CI = 1.4–5.2 μg/mouse) than the functionalised ZnO (BMD10 = 8.5, CI = 4.9–13.1 μg/mouse) due to the lower BMD10 without overlapping CIs (Table 1). Although an increase in the number of macrophages in BALF was detected (Fig 1), no dose-dependent change in the percentage of macrophages was found (Table 1). This demonstrates the importance of analysing both the total number of cells and the percentage of individual cell types to understand the overall inflammatory response. There was no change in the number of eosinophils or lymphocytes (Fig 1A and 1B).

**Table 1:** Summary of BMDs of lung BALF parameters.

	Non-functionalised ZnO *		Functionalised ZnO		Ag
	BMD10	CI	BMD10	CI	BMD10
**Total cell count**	29.5	23.7–55.6	9.4	7.1–64.0	nd
**Neutrophils**	2.8	1.4–5.2	8.5	4.9–13.1	nd
**IL-6**	3.4	2.1–10.0	3.0	1.9–6.4	nd
**GCSF**	nd		3.3	2.0–7.6	nd
**LDH**	19.0	12.2–43.3	nd		nd
**Protein**	17.8	11.5–39.9	1.3	0.6–3.4	nd

All benchmark doses (BMD10, μg/mouse) correspond to 10% increase compared to background levels. CI indicates the 90%-confidence interval of the BMD.

nd—could not be determined, since the data was best described by a horizontal line

* Pooled data

Values for % macrophages, CXCL1 and CCL2 are not included as they were either not detectable or were best described by a horizontal design.

An array of 11 cytokines was analysed in the BALF and presented here in a heat map (Supporting information, S2 Fig). These included pro-inflammatory cytokines known to function as crucial initiators of inflammation (IL1β and TNFα), mediators of acute inflammation and the acute phase response (IL6 and G-CSF) as well as central chemo-attractants for inflammatory cell recruitment, namely for neutrophils (CXCL1) and monocytes (CCL2). Furthermore, Th2 response cytokines, namely IL4 and IL13, were chosen due to their anti-inflammatory and pro-fibrotic activity. IL12, CCL4 and -5 were analysed due to their involvement in a Th1 response that could lead to chronic inflammation. This particular cytokines analysis allowed for the detection of the inflammatory activation of resident macrophages (IL1β, TNFα, and IL12), lymphocytes (IL4, IL13) and non-hematopoietic, epithelial (CXCL1 and CCL4) or endothelial (G-CSF, CCL5) cells of the lung. For all cytokines measured, statistically significant results were only obtained for IL6 and G-CSF for which a BMD10 has been derived (Table 1). The non-functionalised ZnO induced an IL6 response with a BMD10 of 3.4 μg/mouse. For the functionalised ZnO NM, IL6 and GCSF exhibited a very similar BMD10 of 3.0 and 3.3 μg/mouse.

**Fig 2:**
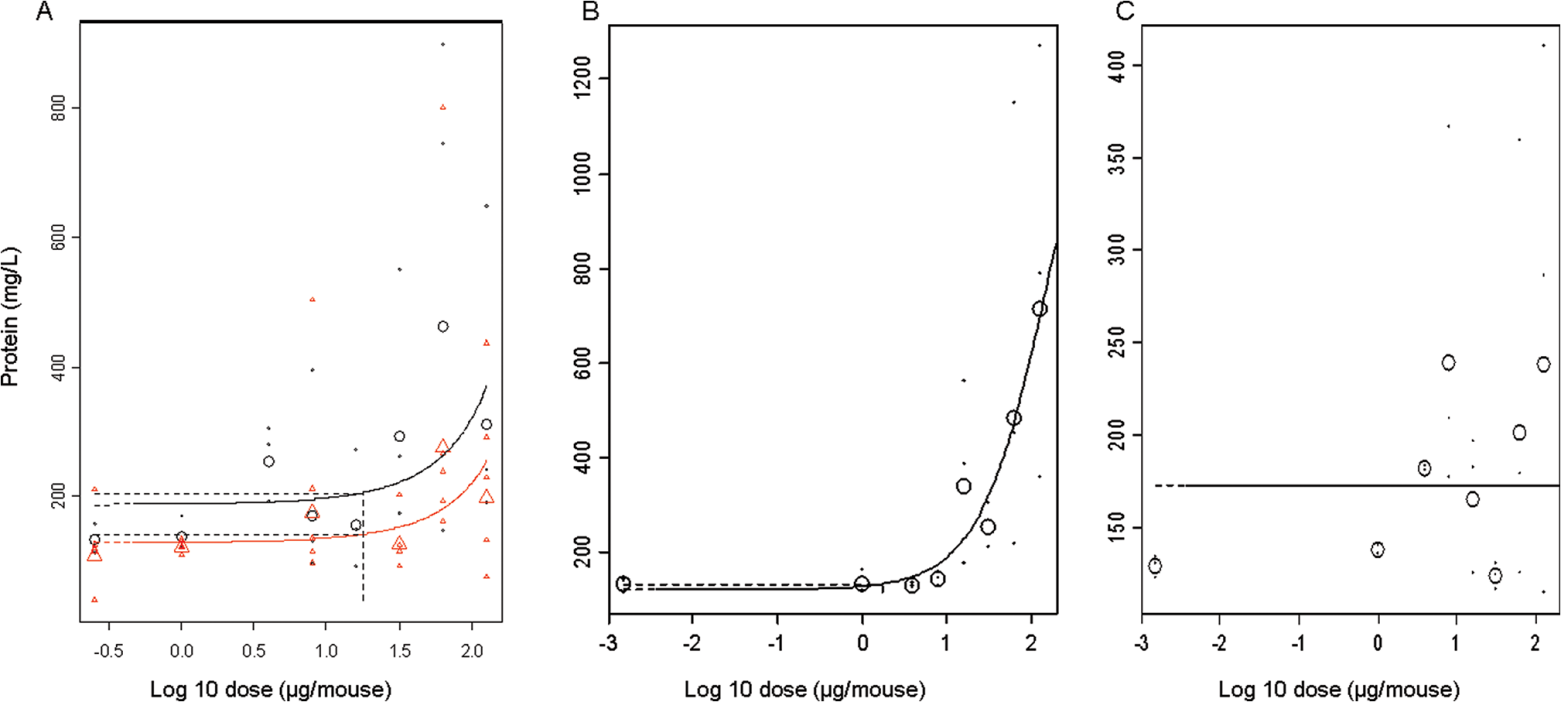
Dose-response relationships for thetotal protein amount in BALF. Dose response relationships were determined for the total amount of protein in the BALF (mg/ml) using benchmark dose modelling. A) two independent administrations of non-functionalised ZnO. The similar shape of the curves allowed pooling the data and deriving a single BMD. B) functionalised ZnO and C) Ag. The large symbols represent the (geometric) mean response of the individual responses (small symbols) in each dose group. The vertical dashed lines represent BMD respectively.

In addition to an inflammatory response, non-functionalised ZnO NMs caused cellular damage in a dose-dependent manner as demonstrated by increases in the level of LDH (BMD10 = 19.0, CI = 12.2–43.3 μg/mouse) (Table 1) and BALF protein content (BMD10 = 17.8, CI = 11.5–39.9 μg/mouse) (Fig 2A). There was no effect on LDH for the functionalised ZnO (Table 1), while a dose-dependent increase in total protein with a BMD10 of 1.3 μg/mouse (CI = 0.6–3.4 μg/mouse) was noted (Fig 2B).

**Fig 3:**
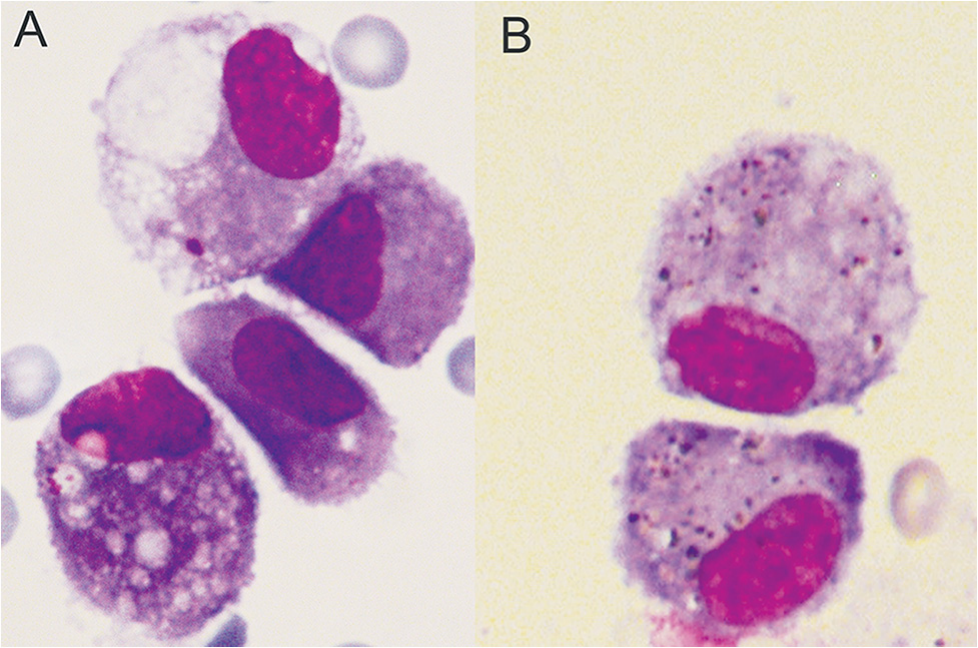
Light microscopic analysis. Images of BALF macrophages 24 hours after administration of A) 16 μg/mouse functionalised ZnO and B) 8 μg/mouse Ag.

In addition, pathological changes in the lung were determined 24 hours after the administration of the vehicle control or non-functionalised ZnO (S1 File). In 2 out of 5 control animals treated with the vehicle control (milliQ water + 2% mouse serum), occasional small focal mononuclear infiltrates were observed (similar to exposed animals). In contrast, desquamated bronchial epithelial cells were observed in 4 out of 5 animals exposed to 64 μg non-functionalised ZnO (representative example in S1 File.) and in 3 out of 5 animals exposed to 128 μg non-functionalised ZnO, with a subsequent neutrophil influx that corresponded to the observed neutrophil influx in the BALF. There were no ZnO NM agglomerates observed inside the macrophages, at any dose utilized in this study (an example of a macrophage from an animal exposed to 16 μg is shown in Fig 3A).

### Acute lung responses—Ag

There was no dose-dependent increase over controls in animals exposed to the Ag NM for any of the lung parameters tested (Fig 1C for cellularity, Fig 2C for total protein, Table 1 for a summary of BMDs and S2 Fig for cytokine response). For most of the dose groups (with the exception of the 1 μg/mouse dose group), silver particle agglomerates were observed inside lavaged macrophages (Fig 3B), but this did not lead to signs of acute cell damage or inflammation.

### Acute systemic responses measured via blood parameters

For the functionalised ZnO, an increase in absolute numbers of blood neutrophils and a decrease in absolute numbers of lymphocytes was observed, indicative of systemic inflammation (Table 2). This was not observed following administration of non-functionalised ZnO. Additionally, only very slight increases in red blood cell numbers, and subsequent increases in haemoglobin levels, were detected following treatment with the non-functionalised ZnO (Table 2). However, the confidence interval around the derived BMD10 value was large and in order to derive a BMD10, extrapolation beyond the highest dose was necessary, reducing the biological relevance of this finding. Similar to non-functionalised ZnO, an increase in haemoglobin and red blood cells was observed following the administration of functionalised ZnO. However, these effects were more prominent, since a 10% increase was observed at a lower BMD10 compared to controls. The administration of non-functionalised ZnO also led to the increase in total number of platelets which was not observed for the functionalised ZnO NM.

**Table 2:** Summary of benchmark doses (BMDs) of blood parameters.

	Non-functionalised ZnO *		Functionalised ZnO		Ag	
	BMD10	CI	BMD10	CI	BMD10	CI
**Number of neutrophils**	nd		14.2	9.0–34.3	nd	
**Number of lymphocytes**	nd		23.4	12.8–130	nd	
**Number of red blood cells**	194 #	115–604.1	119.0#	66.4–580	215 #	126–733
**Haemoglobin**	230 #	126.0–1299	97. #	58.0–311	nd	
**Platelets**	72.5	53.2–114	nd		nd	

All benchmark doses (BMD10, μg/mouse) correspond to 10% increase compared to background levels. CI indicates the 90%-confidence interval of the BMD.

nd—no dose-response could be fitted

* Pooled data

# Extrapolation of point estimate or confidence interval of BMD beyond the highest dose administered

There were no significant effects on any blood parameter following exposure to the Ag NM with the exception of a slight increase in red blood cell numbers at a benchmark dose extrapolated beyond the highest administered dose (Table 2).

### Effects in the liver

**Fig 4:**
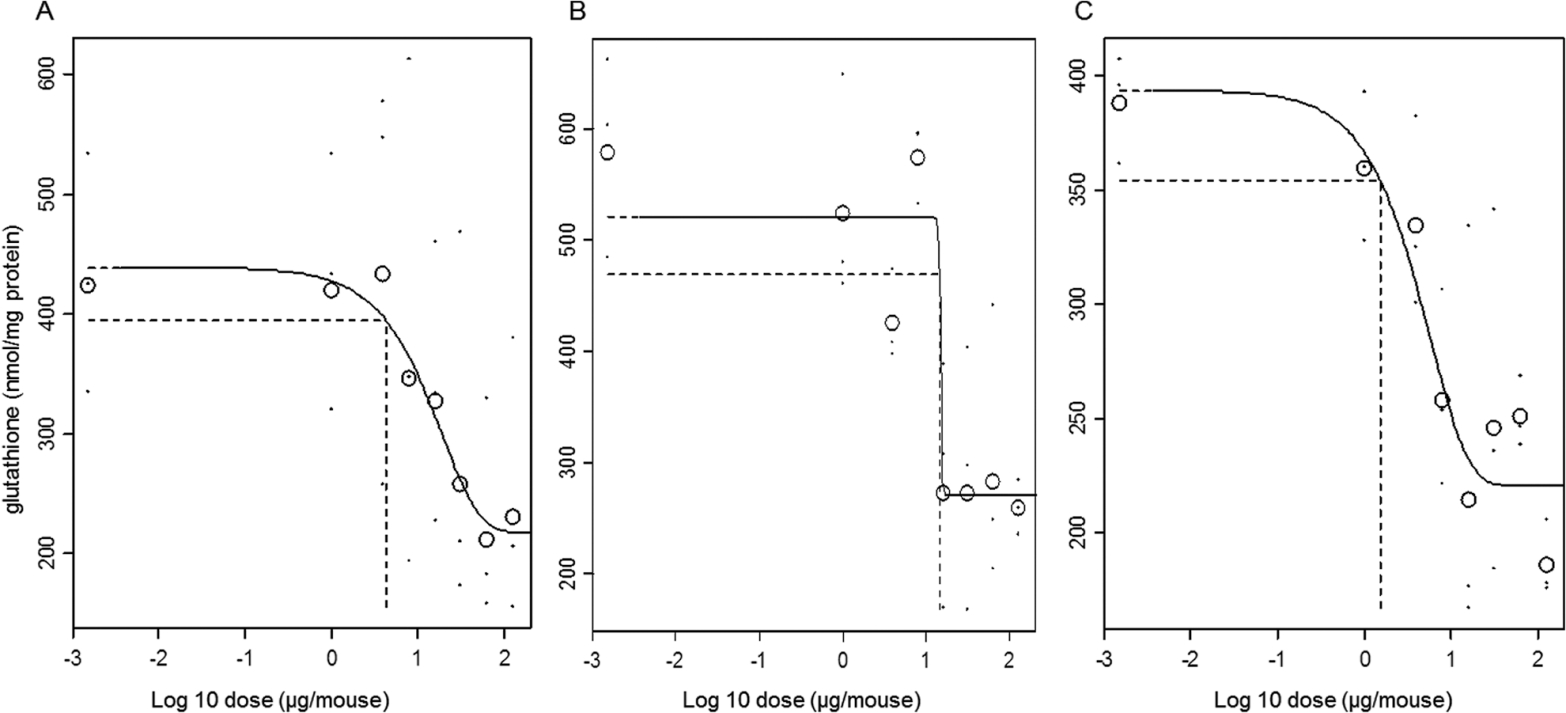
Dose-response relationships for glutathione depletion in the liver. A) non-functionalised ZnO, B) functionalised ZnO and C) Ag NMs. The large symbols represent the (geometric) mean response of the individual responses (small symbols) in each dose group. The horizontal and vertical dashed lines represent the BMR and BMD respectively.

Dose-dependent decreases in glutathione levels were detected compared to vehicle control animals after exposure to non-functionalised ZnO, functionalised ZnO and Ag NMs (Fig 4). The Ag NM was the most potent in reducing glutathione levels based on the lowest derived BMD10 (1.5 μg/mouse, CI = 0.7–3.6 μg/mouse) followed by non-functionalised ZnO (4.2 μg/mouse, CI = 1.4–15.1 μg/mouse) and functionalised ZnO (15.6 μg/mouse, CI = 7.7–15.7 μg/mouse). We suspected that this decrease could be associated with the presence of Ag in the tissue itself. Therefore, we determined total Ag levels in the liver by HR-ICPMS in vehicle control mice and the two highest dose groups (summarized in S3 Table). In vehicle control mice, the amount of Ag was below the detection limit (< 0.01 μg/g tissue). In mice that received 64 or 128 μg/mouse, significant amounts of silver were detected (0.02 ± 0.001 and 0.49 ± 0.78 μg/g tissue respectively). In one mouse in the 128 μg/mouse dose group, very high Ag levels were detected of 1.4 μg/g tissue, explaining the rather large standard deviation.

### Summary for acute responses

**Fig 5:**
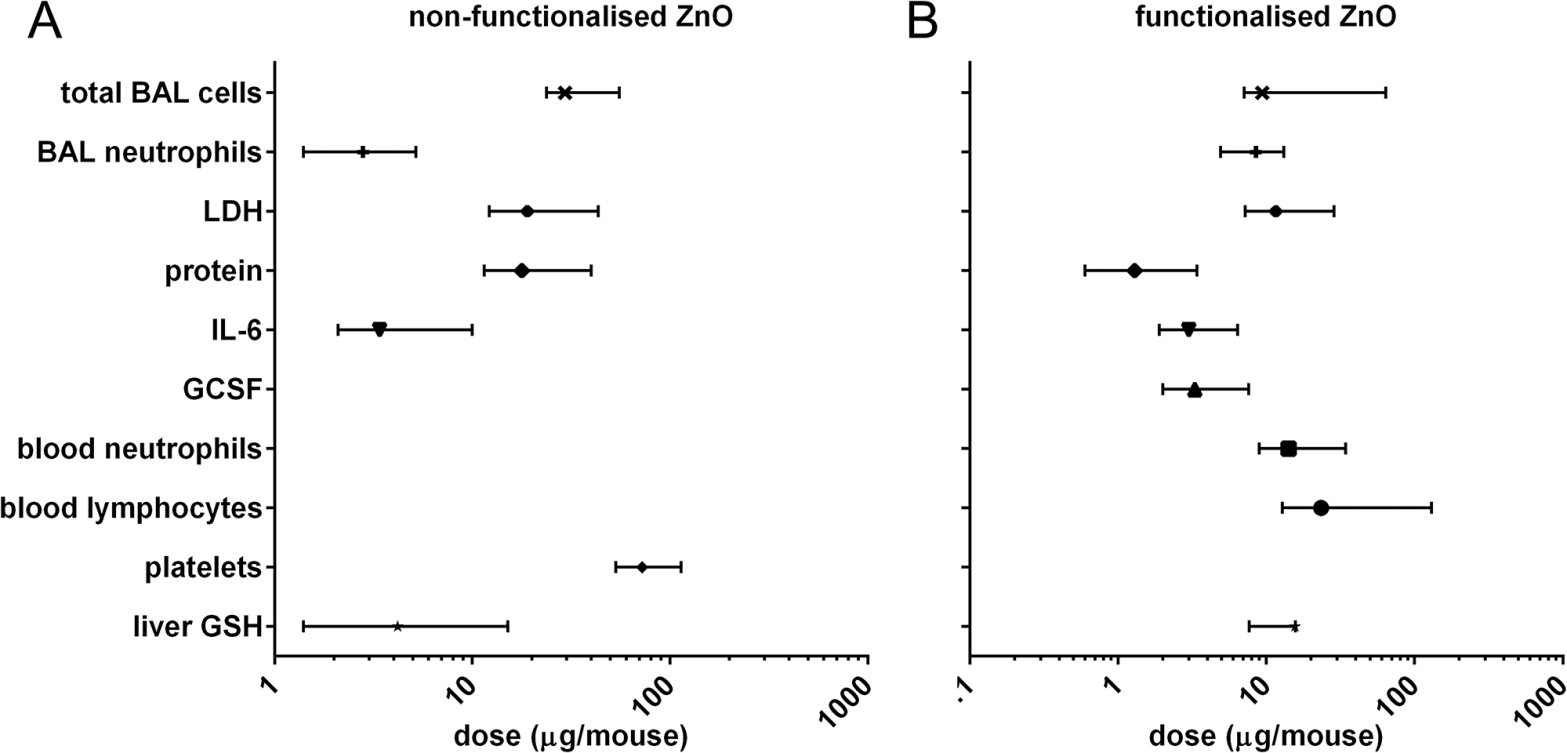
Summary of benchmark dose relationships. A) non-functionalised ZnO and B) functionalised ZnO (Log scale on the x-axis). BMDs including the confidence interval are plotted for those endpoints for which an effect was found.

For both ZnO NMs, increases in the neutrophil numbers (PMN) and IL-6 levels in the BALF were noted as sensitive markers for lung inflammation. Experimental error and biological variation have a low impact on these endpoints given the small confidence intervals around the BMD10 (Fig 5). The cell damage marker “total protein” was the most sensitive marker for functionalised ZnO exposure. For non-functionalised ZnO similar less significant effects were observed for the cell damage markers LDH and total protein (Fig 5).

With respect to the systemic endpoints such as recruitment of inflammatory cells into the bloodstream, statistically significant changes were found at higher instilled doses. This might be due to the fact that only a small fraction of the instilled dose became systemically available. It is therefore surprising that a 10% decrease in glutathione levels in the liver was observed even at a relatively low dose for both types of ZnO as well as Ag. The presence of Ag in the liver tissue itself was detected and the liver glutathione response was observed in the absence of measured effects in the lung or other systemic responses.

## Discussion

The current study aimed to investigate *in vivo* toxicity of three NMs, a functionalised ZnO, a non-functionalised ZnO and an Ag NM, all of which have been demonstrated to impact on cell viability *in vitro* compared to other NMs such as TiO_2_ and MWCNTs [4] (S1 Fig). A pulmonary inflammatory response with cell damage was observed 24 hours after I.T. instillation of both non-functionalised and functionalised ZnO NMs. Previously, a similar response has been demonstrated after a single comparable dose of ZnO nanoparticles in rats [30]. In humans, exposure to zinc fumes (ZnO) from welding, cutting, or brazing galvanized metal can cause metal fume fever [31] and an increase in the number of pro-inflammatory cytokines and neutrophils in BALF have also been observed in a controlled clinical experiment [32].

With respect to systemic effects induced by the functionalised and non-functionalised ZnO NM exposure, the observed increased IL-6 in blood reflects the symptoms of metal fume fever [33]. In rat and mouse studies, ZnO nanomaterials have induced both lung and systemic inflammation [34, 35]. Here we observed an increase in blood neutrophils and a decrease in blood lymphocytes indicative of an inflammatory response following the administration of the functionalised ZnO. However, this was not observed for non-functionalised ZnO NM. The reason for this difference is unknown. The solubility of the ZnO NMs in water and cell culture medium have been reported to be similar [4]. However, it is important to note that the relative solubility or biopersistence of the two ZnO NMs *in vivo* is not known. It is also not known if the triethoxycaprylylsilane could be released from the nanomaterial over time and be responsible for an enhanced inflammatory response in the lung. We hypothesise that the functionalised NM could be more stable allowing for longer persistence in the body and increased inflammatory responses.

For Ag NMs, no acute pulmonary inflammation, lung cell damage or systemic inflammation was observed up to a dose of 128 μg/mouse (6.4 mg/kg bw). In literature, exposure to Ag NMs has resulted in different degrees of pulmonary responses. These vary from no inflammation at all after inhalation of 3.5 μg/m^3^ (6 hours per day, 5 days a week for 28 days resulting in a dose of 1.3 μg/rat) [36] or after instillation of 10 μg/mouse [37], to mild inflammation and cytotoxicity after inhalation of 3.3 mg/m³ (with a total deposited dose of about 60 μg and a retained dose after the end of the last exposure of around 4 μg/mouse) [38] or an instillation of 17.5 mg/kg bw in rats [39]. These materials, from different suppliers or generation methods, are likely to differ in their physico-chemical characteristics in addition to differences in their primary particle size and could explain the wide range of biological effects.

### Mechanism of toxicity

The mechanism of toxicity for ZnO and Ag NMs, involves the solubility of the particles and the subsequent release of ions [40–43]. ZnO has a relatively low solubility in water, but pH is known to influence this [44]. The slightly acidic nature of the lung lining fluid or even the more acidic milieu inside a lysosome of a lung macrophage could lead to a local increase in zinc ion generation [45]. For larger particles at low pH (pH 2.7), a high solubility was indicated in the SCCS opinion (89.6% solubility for particles >3mm, 98.5% for particles <1mm) [46]. It has been demonstrated that ZnO nanoparticles (from a different supplier than presented here) are rapidly dissolved under acidic conditions (pH 4.5) whilst remaining intact at pH 7.4 [47]. We did not observe any particulate material inside macrophages 24 hours after ZnO administration using light microscopy, while in a freshly prepared dispersion of ZnO particles in water (in the absence of cells) agglomerates were visible. This suggests substantial dissolution within the 24 hour time period after in vivo administration. Dissolution of the same material was also found in a study using human hepatocytes *in vitro*, after Transmission Electron Microscopy analysis [48]. Cell death of primary and human hepatocytes was observed, while no Zn nanoparticles were detected inside the cell suggesting complete dissolution.

After administration of Ag NM, agglomerates were observed by light microscopy inside the lung macrophages after 24 hours. Ag particles may need a longer time to dissolve compared to ZnO particles *in vivo*, as has been observed *in vitro* in cell culture medium and water [4]. In these experiments, the same Ag NM as used here had a very low solubility in water (7 10^–6^ and 6.3 10^–4^ mg/ml at 1 and 128 mg/ml, respectively) and even lower solubility in cell culture medium (1 10^–6^ and 3.9 10^–4^ mg/m at 1 and 128 mg/ml, respectively). Dissolution studies with a 5 nm Ag aerosol of a different source, showed that particles did not dissolve in a solution mimicking an extracellular (Gamble’s solution pH 7.4) or intracellular milieu (Artificial Lysosomal Fluid, ALF, pH 4.5) within a 24 hour period [38]. Similar studies with other commercially available Ag NMs showed no detectable dissolution within 96 hours [49]. Unfortunately, we have no 24 hour dissolution data of the particular Ag NM with its specific coating used here in (artificial) lung lining fluid or a solution mimicking the intracellular macrophage environment with a low pH. In any future study, it would be interesting to determine this solubility to see whether it could predict the pulmonary and systemic responses. The use of (enriched) stable isotopes *in vivo* would also be useful to understand the bio distribution of the ZnO NMs with and without functionalisation.

### Liver responses

With respect to distal endpoints, exposure to the Ag NM resulted in the depletion of glutathione in the liver. This would suggest that Ag NMs or Ag^+^ ions became systemically available, since silver could be detected in liver tissue by HR-ICP-MS. This could provide an explanation for the observed anti-oxidant depletion. The type of detection applied here does not discriminate between a particulate or ionic form after the dissolution of silver. However, given the short time frame for the exposure (24 hours), the slow dissolution of Ag in pulmonary macrophage lysosomes and the evidence that other particles (such as gold or iridium) can translocate from the lung [6, 11, 12], we speculate that it may be the particulate form. While minute amounts of Ag might become available through the alveolar blood barrier, significant levels are also likely to reach the liver after mucociliary clearance following uptake in the GI-tract [8]. The effects observed here could be related to the translocation of NMs, adsorption of ions to the liver or rather because of inflammatory mediators released in the circulatory system following exposure to the NMs or as a result of a combination of these factors. Interestingly, similar depletion in glutathione levels has been reported after exposure of C3A liver cells *in vitro* to these three materials [50], which confirms that Ag can have a direct effect on hepatocytes, the cell type constituting about 80% of the liver tissue. Also other recent studies indicate that Ag uptake via the GI-tract can challenge the liver via oxidative stress dependent pathways [51–53]. It is important to note that we do not associate these anti-oxidant alterations with acute toxicity in the liver, but more with a coping mechanism following oxidative stress. Overall, it is interesting to see distal effects of these NMs at relatively low doses, especially in the absence of a local lung effect in the case of the Ag NM.

### Comparison to *in vitro* results

In collaboration with this study, previous *in vitro* studies have demonstrated the cytotoxicity of both types of ZnO as well as Ag NMs in hepatocytes and renal cells [4, 5]. We have also observed the ability of ZnO and Ag NMs to induce dose dependent cytotoxicity in a cell model relevant to lung administration (S1 Fig). An IC_50_ of 10 μg/cm^2^ was noted for the murine alveolar lung epithelial cell line (LA-4) for the three NMs utilised in this study after a 24 hour exposure. A more varied pattern was observed for MH-S alveolar macrophages with IC_50_ values of 5, 10 and 20 μg/cm^2^ for the Ag, non-functionalised and functionalized ZnO NM respectively, indicating that Ag NMs are more toxic to these phagocytes. In addition, there appears to be a minor protective effect of the functionalization for ZnO (S1 Fig). Besides cytotoxic effects, an increase in inflammatory gene expression at doses of 0.7 and 2.5 μg/cm^2^ ZnO (or 0.09 and 0.33 cm^2^ ZnO/cm^2^) in A549 alveolar epithelial cells has also been stated [54]. Additionally, seven other NMs were investigated in the same project including a number of TiO_2_ NMs and multi-walled carbon nanotubes. Since these NMs showed no significant toxicity *in vitro* at doses up to 118 μg/cm^2^, they were not investigated further *in vivo*.

In contrast to the *in vitro* studies, we only observed an inflammatory response and cytotoxicity in the lung for the ZnO NMs (not for Ag) *in vivo*. The reasons for these differences are not clear, but could be due to the different dissolution dynamics of the materials *in vitro* compared to *in vivo* or to a different protection mechanism in the mouse lung that reduces the cytotoxicity of Ag. Another explanation might arise from a fast cleared AgNP fraction into the GI-tract that might succumb to its special milieu leading to different dissolution kinetics.

### Hazard identification and ranking

Benchmark dose modelling allowed for hazard identification based on acute inflammation and cell damage endpoints of the three materials. The triethoxycaprylylsilane functionalized ZnO ranked as the most potent NM based on the largest number of affected endpoints, as well as the strongest responses observed, followed by the non-functionalised ZnO material. The Ag NM was the least potent material resulting only in reduced glutathione levels in the liver. Remarkably, this systemic effect was observed in the absence of noticeable effects in the lung. Although the three nanomaterials differ in primary particle size, the agglomerate size in the dispersion medium is similar (around 200 nm). Therefore, we believe that differences in particle size are not a major influence on the ranking of the toxicity. With the current study design, we realize that only a ranking of the acute hazard of the three materials can be made based on the administered mass. Our study design and subsequent statistical analysis allows for multiple endpoint determination in the lung as well as other organs to identify sensitive endpoints for further study, either by expanding the set of NMs tested or as a prioritisation tool for e.g. more elaborate inhalation studies.

## Supporting Information

S1 Dataset (XLS)

S1 Fig Cell viability by WST-1 assay. A) LA-4 and B) MH-S cells 24 hours after incubation with ten different nanomaterials such as two types of MWCNTs (NM-400 and NM-402), two types of zinc oxide (non-functionalised NM-110 and functionalised NM-111), one type of silver (NM-300) and 5 types of TiO_2_ (NRCWE001, NRCWE-002, NRCWE-003, NRCWE-004 and NM-101). Membrane damage was assessed by LDH release in C) LA-4 and D) MH-S cells 24 hours after incubation with the same nanomaterials except for NM-300.
(DOCX)

S2 Fig Heat map of cytokine release in BALF. The data is analysed with Luminex after ZnO NM-110, ZnO NM-111 and Ag NM-300 instillation. The results are expressed in fold increase over control for the vehicle control group (VC) and the 5 highest dose-groups.
(DOCX)

S1 File Histopathology of mouse lungs. (PDF)

S1 Supplementary Materials and Methods (DOCX)

S1 Table Nanomaterial characteristics. (DOCX)

S2 Table Particle size distribution in suspension as determined by particle tracking analysis. (DOCX)

S3 Table Silver content in the liver determined by HR-ICPMS. (DOCX)
