# Supplementary material for: Comparative Hazard Identification by a Single Dose Lung Exposure of Zinc Oxide and Silver Nanomaterials in Mice
Source: PLoS One. 2015 May 12;10(5):e0126934. doi: 10.1371/journal.pone.0126934 (PMC4429007; doi:10.1371/journal.pone.0126934)
Supplement: S1 Fig — A) LA-4 and B) MH-S cells 24 hours after incubation with ten different nanomaterials such as two types of MWCNTs (NM-400 and NM-402), two types of zinc oxide (non-functionalised NM-110 and functionalised NM-111), one type of silver (NM-300) and 5 types of TiO2 (NRCWE001, NRCWE-002, NRCWE-003, NRCWE-004 and NM-101). Membrane damage was assessed by LDH release in C) LA-4 and D) MH-S cells 24 hours after incubation with the same nanomaterials except for NM-300. (DOCX) [file pone.0126934.s002.docx]

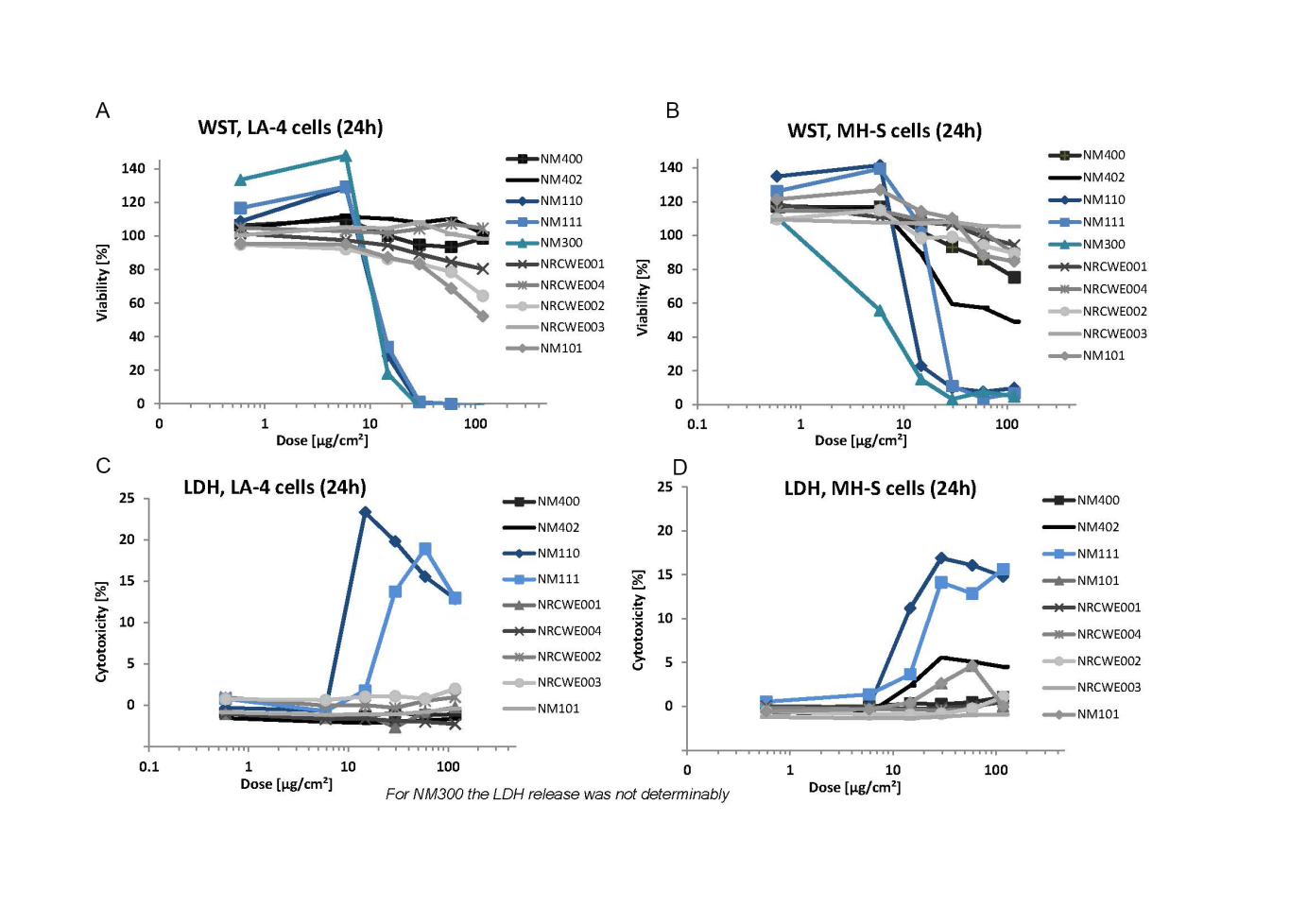


**S1 Fig. Cell viability by WST-1 assay.** A) LA-4 and B) MH-S cells 24 hours after incubation with ten different nanomaterials such as two types of MWCNTs (NM-400 and NM-402), two types of zinc oxide (non-functionalised NM-110 and functionalised NM-111), one type of silver (NM-300) and 5 types of TiO_2_ (NRCWE001, NRCWE-002, NRCWE-003, NRCWE-004 and NM-101). Membrane damage was assessed by LDH release in C) LA-4 and D) MH-S cells 24 hours after incubation with the same nanomaterials except for NM-300.
