# Supplementary material for: Comparative Hazard Identification by a Single Dose Lung Exposure of Zinc Oxide and Silver Nanomaterials in Mice
Source: PLoS One. 2015 May 12;10(5):e0126934. doi: 10.1371/journal.pone.0126934 (PMC4429007; doi:10.1371/journal.pone.0126934)
Supplement: S2 Fig — The data is analysed with Luminex after ZnO NM-110, ZnO NM-111 and Ag NM-300 instillation. The results are expressed in fold increase over control for the vehicle control group (VC) and the 5 highest dose-groups. (DOCX) [file pone.0126934.s003.docx]

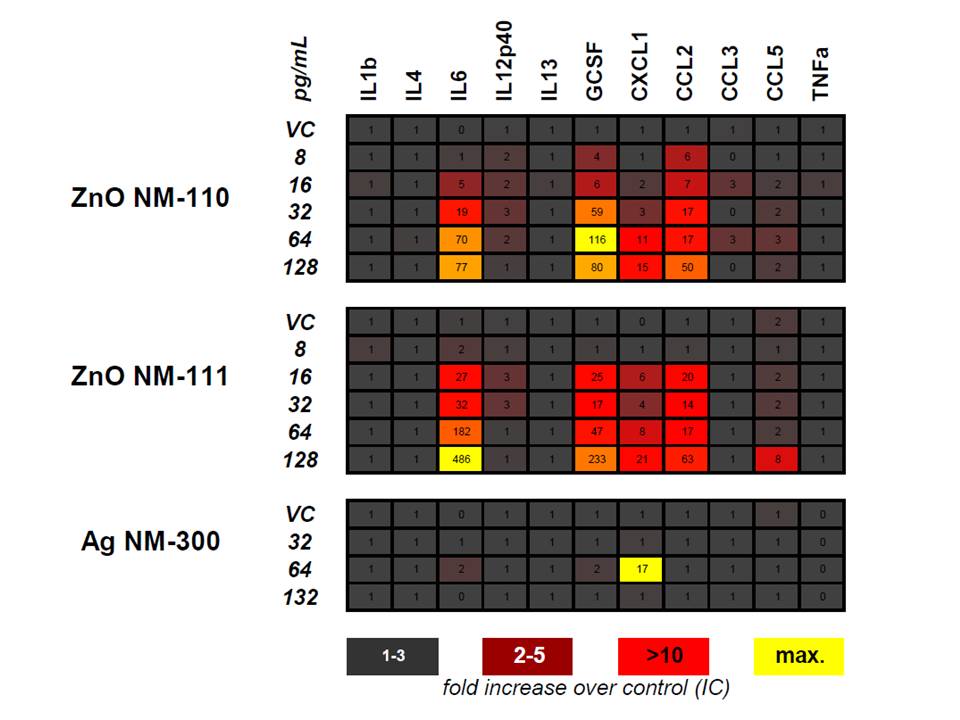


**Nano Ag**

**Non- functionalised ZnO**

**Functionalised ZnO**

**S2 Fig. Heat map of cytokine release in BALF**. The data is analysed with Luminex after ZnO NM-110, ZnO NM-111 and Ag NM-300 instillation. The results are expressed in fold increase over control for the vehicle control group (VC) and the 5 highest dose-groups.
