# Supplementary material for: Comparative Hazard Identification by a Single Dose Lung Exposure of Zinc Oxide and Silver Nanomaterials in Mice
Source: PLoS One. 2015 May 12;10(5):e0126934. doi: 10.1371/journal.pone.0126934 (PMC4429007; doi:10.1371/journal.pone.0126934)
Supplement: S1 File — (PDF) [file pone.0126934.s004.pdf]

S1 File. Histopathology of mouse lungs.

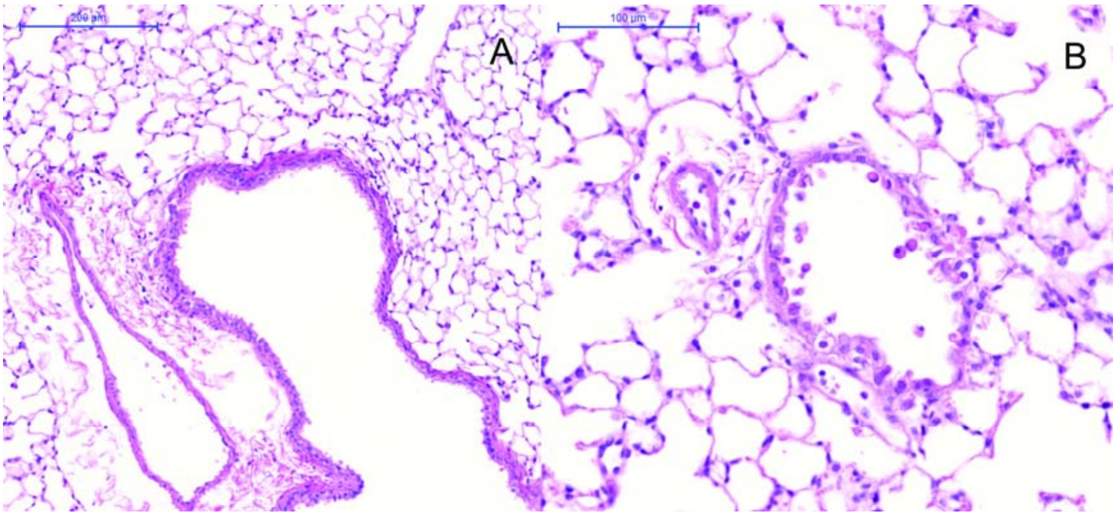

Figure 1 Photograph A: Visualisation of mouse lungs 24 hours after administration of vehicle control of non-functionalised ZnO, 10x magnification. Photograph B: 64 µg/mouse non-functionalised ZnO showing desquamation of bronchiolar epithelial cells, 20x magnification.

Table 1 Histopathology in the mouse lung after administration of non-functionalised ZnO

	**0 mg/mouse**<br>Number of mice (total)	**64 mg/mouse**<br>Number of mice (total)	**128 mg/mouse**<br>Number of mice (total)
Desquamation of bronchiolar epithelial cells	0 (5)	4 (5)	3 (5)
