# Supplementary material for: Comparative Hazard Identification by a Single Dose Lung Exposure of Zinc Oxide and Silver Nanomaterials in Mice
Source: PLoS One. 2015 May 12;10(5):e0126934. doi: 10.1371/journal.pone.0126934 (PMC4429007; doi:10.1371/journal.pone.0126934)
Supplement: S1 Supplementary Materials and Methods — (DOCX) [file pone.0126934.s005.docx]

**Supplementary Materials & Methods**

Nanomaterials used for *in vitro* testing

Ten different nanomaterials were tested in vitro, namely two types of multiwall carbon nanotubes (MWCNTs) NM-400 (entangled MWCNT, diameter 30 nm) and NM-402 (entangled MWCNT, diameter 30 nm), two types of zinc oxide NM-110 (zinkite, non-functionalised, 100 nm), NM-111 (zinkite functionalised with triethoxycaprylylsilane, 130 nm), one type of silver NM-300 (Ag capped with polyoxylaurat Tween-20 <20 nm) and 5 types of titanium dioxide NRCWE-001 (TiO_2_ rutile 10 nm NanoAmor, Houston, TX, USA), NRCWE-002 (TiO_2_ rutile 10 nm with positive charge), NRCWE-003 (TiO_2_ rutile 10 nm with negative charge), NRCWE-004 (TiO_2_ rutile 94 nm, NaBond Technologies Co.,China), NM-101 (rutile with minor anatase; 7 nm), The above-mentioned NMs were subsampled and preserved under argon in the dark until use. The NMs were received from the European Commission Joint Research Centre (Ispra, Italy). Details on the characterization are described previously [1].

*In vitro* assays of viability (WST-1) and cytotoxicity (LDH)

For the viability and cytotoxicity assays we used MH-S cells, a murine alveolar macrophage cell line grown in DMEM (dulbecco´s MEM) (Biochrom/FG0435) and 10 % FBS (Biochrom/SO615), and LA-4 cells, a murine lung-epithelial like cell line grown in HAM´s F12 (Biochrom/FG0815) plus NEA (non-essential amino acids, Biochrom/K0293) and 15 % FBS (Biochrom/SO615). Cells were seeded at 2.5 x 10^4^ cells in 200µl per well in a 96 well plate and incubated over-night to obtain 90% confluency. The NMs were resuspended according to the sonication protocol as described in the Materials & Methods section of the main paper in LiChrosolv water/2% FCS at a stock concentration of 2.56 mg/ml. Dilutions from this stock suspensions were made to obtain final concentrations of 0, 0.6, 5.9, 14.7, 29.4, 58.8, and 117.6 µg/cm^2^ in 200µl/well. At the end of the exposure after 24 hours, the supernatants were removed and aliquots of 100 µl were kept for LDH testing and stored over-night at 4°C. Cell viability was measured using the cell proliferation reagent WST-1 (Roche Applied Sciences, Cat. No. 11 644 807 001, Germany). A volume of 7.5 µl WST-1 (5%) (Roche Diagnostics) was added to 150µl fresh medium per well and incubated for 15 min at room temperature. The plate was shaken thoroughly for 1 minute, the supernatants were centrifuged and transferred to a new plate to measure the optical density at 450 nm in a standard plate reader (Labsystems iEMS Reader MF, Finland).

Cytotoxicity was assessed by detection of the enzyme lactate dehydrogenase (LDH) in the supernatant of the cell culture (see above). Leakage of this enzyme from the cytoplasma into the supernatant is characteristic of membrane damage. The assay was performed using the Cytotoxicity Detection Kit (Roche Diagnostics, Cat. No. 11 644 793 001, Mannheim, Germany) according to the manufacturer’s protocol. The LDH concentration in 100 µl of the cell culture supernatant was determined at a wavelength of 492 nm. Cells treated with 2 % (w/v) Triton X-100 served as reference for the maximum possible LDH release (100%, high control). The relative LDH release of a given sample is then defined as the ratio of LDH measured in the supernatant of the sample and the high control value with LDH values under 10% regarded as a nontoxic effect level.

**Reference**

1. Kermanizadeh A, Pojana G, Gaiser BK, et al. (2013) In vitro assessment of engineered nanomaterials using a hepatocyte cell line: cytotoxicity, pro-inflammatory cytokines and functional markers. Nanotoxicology 7:301-13
