# Supplementary material for: Comparative Hazard Identification by a Single Dose Lung Exposure of Zinc Oxide and Silver Nanomaterials in Mice
Source: PLoS One. 2015 May 12;10(5):e0126934. doi: 10.1371/journal.pone.0126934 (PMC4429007; doi:10.1371/journal.pone.0126934)
Supplement: S1 Table — (DOCX) [file pone.0126934.s006.docx]

**S1 Table.** **Nanomaterial characteristics**

|  |  | Particle size  (nm) | | | Surface | |
| --- | --- | --- | --- | --- | --- | --- |
|  | **Sample** | **Manufacturer** | **XRD** | **TEM** | **BET (m2/g)** | **coating** |
| Non-function. ZnO | NM-110 | 100 | 70 to > 100 | 20-250/50-350 | 14 | none |
| Functionalised ZnO | NM-111 | 130 | 58-93 | 20-200/10-450 | 18 | Triethoxycaprylylsilane 130 |
| Nano Ag | NM-300 | <20 | 7* or 14# | 8–47 | NA | Polyoxylaurat Tween 20 |

* Wet XRD in capillary tube

# Dried samples

NA not applicable

Ref (Kermanizadeh et al. 2013)
