# Supplementary material for: Comparative Hazard Identification by a Single Dose Lung Exposure of Zinc Oxide and Silver Nanomaterials in Mice
Source: PLoS One. 2015 May 12;10(5):e0126934. doi: 10.1371/journal.pone.0126934 (PMC4429007; doi:10.1371/journal.pone.0126934)
Supplement: S2 Table — (DOCX) [file pone.0126934.s007.docx]

**S2 Table.** **Particle size distribution in suspension as determined by particle tracking analysis**

|  |  | Particle size distribution in suspension (nm) | |
| --- | --- | --- | --- |
|  | **Sample code** | **Median (+/- sd)** | **Mean (+/- sd)** |
| Non-functionalised ZnO | NM-110 | 235 +/- 42 | 223 +/- 66 |
| Functionalised ZnO | NM-111 | 208 +/- 74 | 225 +/- 32 |
| Nano Ag | NM-300 | 234 +/- 77 | 155 +/- 25 |
