# Supplementary material for: Comparative Hazard Identification by a Single Dose Lung Exposure of Zinc Oxide and Silver Nanomaterials in Mice
Source: PLoS One. 2015 May 12;10(5):e0126934. doi: 10.1371/journal.pone.0126934 (PMC4429007; doi:10.1371/journal.pone.0126934)
Supplement: S3 Table — (DOCX) [file pone.0126934.s008.docx]

**S3 Table. Silver content in the liver determined by HR-ICPMS**

| Treatment | Silver content [μg/g tissue] |
| --- | --- |
| Vehicle control (n=3) | all: <0.01 |
| 64 µg Ag as NM-300 /mouse (n=3) | 0.02 ± 0.01  Individual results:  0.01; 0.03; 0.01 |
| 128 µg Ag as NM-300 /mouse (n=3) | 0.49 ± 0.79  Individual results:  0.04; 1.49; 0.04 |
